# Supporting adolescents with self-harm: practitioners’ experiences of delivering a rapid brief psychological intervention to adolescents presenting to Emergency Departments

**DOI:** 10.3389/frcha.2026.1959051

**Published:** 2026-10-14

**Authors:** Sally O’Keeffe, Maria Long, Tania Saour, Rose McCabe

**Affiliations:** 1 Population Health Sciences Institute, Newcastle University, Newcastle-Upon-Tyne, United Kingdom; 2 Department of Population Health and Policy, City St George’s University of London, London, United Kingdom; 3 West London NHS Trust, London, United Kingdom

**Keywords:** adolescent, Emergency Department, safety planning, self-harm, Self-Injurious Behaviour, Solution-Focused Brief Therapy, suicide, therapeutic assessment

## Abstract

**Background:**

The risk of suicide is increased amongst young people following attendance at the Emergency Department (ED) for self-harm. Evidence is limited for effective interventions. Early intervention is crucial before self-harm becomes established as a coping mechanism. The Supporting Adolescents with Self-Harm (SASH) intervention focuses on supporting young peoples’ agency and resources—comprising Therapeutic Assessment, Enhanced Safety Planning and Solution-Focused Follow-Up sessions—and was evaluated in a randomised controlled trial.

**Aim:**

To explore the experiences of CAMHS crisis team practitioners delivering the SASH intervention to young people presenting to EDs with self-harm in the SASH trial.

**Methods:**

The trial assessed the effectiveness of the SASH intervention. This study forms part of the qualitative process evaluation exploring the experiences of practitioners delivering the intervention. Semi-structured interviews were conducted with 15 practitioners who delivered the intervention and four senior staff members. Interviews were analysed using thematic analysis.

**Results:**

Six overarching themes were identified: (1) A transformation in practice, bridging the gap in crisis care by offering an immediate therapeutic intervention and time to build rapport and work with the system around young people; (2) Patient-centred care that builds shared understanding of the reasons underlying self-harm between the young person, carer and practitioner; (3) Harnessing young people's strengths and motivating change; (4) Carer involvement in individual/joint sessions leads to better outcomes when the young person wants them involved and they are available; (5) SASH worked well for most young people, although it may be less suited to those who are less able to express themselves, or have complex difficulties or difficulties that are outside their control; and (6) At first, a paradigm shift for practitioners from focusing on problems to solutions, but over time practitioners gained confidence and motivation in using the SASH intervention and continue to use it in their practice.

**Discussion:**

The SASH intervention offers continuity of care after ED attendance for young people following a self-harm crisis by focusing on building shared understanding of the underlying reasons for self-harm and harnessing their strengths, bridging the gap in crisis care. Supporting carers may also improve outcomes for young people.

**Clinical Trial Registration:**

https://www.isrctn.com/, identifier ISRCTN81846131.

## Introduction

Self-harm refers to any intentional act of self-injury or self-poisoning, irrespective of the motivation (1). Self-harm is common, with one quarter of 17-year-olds in the UK estimated to have self-harmed in the past 12 months (2). Rates of self-harm have increased over recent years in the UK (3), and globally the estimated prevalence of self-harm in adolescents is 16.9%, based on studies of young people aged 12–18 years (4). Self-harm is the strongest risk factor for suicide in adolescence (5), which is the leading cause of death in young people aged 15–24 years in the UK (6). Half of young people aged 10–19 years who die by suicide in the UK have a history of self-harm (7). Among adolescents aged 10–18 years old, the incidence of suicide in the 12 months after self-harm is over 30 times higher than the rate in the general population in the UK (8). This makes adolescence a critical period for early intervention, yet a recent meta-analysis found no therapeutic intervention for self-harm to have a significant impact on future mortality in adolescents (9).

**Table 1:** Quotes for theme 1.

Theme 1: A transformation in practice, bridging the gap in crisis care by offering an immediate therapeutic intervention and time to build rapport, and an opportunity to work with the system around young people
Quote 1a: I thought it was beneficial for the young people, especially with regards to bridging the gap in the provision between having left A&E and moving on to another team because of the wait lists. So sometimes. So, for example, if we're going to refer [to community CAMHS], that that was very helpful in bridging that gap. (Practitioner 3309)
Quote 1b: I like to look at all the factors, all the involved parties and views. And sometimes that's not possible in crisis work, but through the SASH intervention and having the six sessions with her, I was able to expand a bit as well and work with the system, not just working on herself harming behaviours, but also be going a bit beyond. (Practitioner 1020)
Quote 1c: And like there was a time where I described it like drawing blood from a stone because you just felt like the young person just was not engaged at all, and it felt like obviously without the engagement, it was a one-sided conversation and it just meant that, you know, you couldn't progress. It took a few sessions to kind of break down those barriers. Obviously, we got there in the end, but it I think it's just a time thing because even like with my most recent young person, I think all in all I did 6 sessions with her, but genuinely I didn't crack her shell until like the 4th or 5th session, so it took us such a long time to get there. So, I think again, it's that persistence. (Practitioner 2009)
Quote 1d: And the other thing that we picked up was more about the wider needs for the young people, so some of the challenges may have been missed [in usual care] due to the of the urgency or the crisis presentation and the crisis intervention, but when it came to SASH, we had a better understanding of what the need was with the [therapeutic] assessment that we had, the enhanced safety plan and the follow-up sessions. So, we felt that young people are more kind of like settled and more open with regards to what they were struggling with. It made our referrals on better- more informed. (Practitioner 3308)
Quote 1e: And it doesn't always have to be in depth therapy. In the first instance it can be quite simple, without overwhelming them. (Practitioner 1020)
Quote 1f: [Practitioners] can see a change in young people, and they can feel that they are providing some treatment, rather than just managing reactively. At the same time, the young people and the families can also feel better supported because the continuity of care is that the people who see them in the crisis is providing the treatment and they don't need to be repeating themselves constantly. That's one of the things that young people and families normally complain about—that they have to be repeating the same information to different people all the time, so it stays in the same place. And maybe getting better outcomes. And for CAMHS actually reducing the waiting list load that is normally high. (Practitioner 2002)

**Table 2:** Quotes for theme 2.

Theme 2: Building shared understanding between the young person, carer and practitioner
Quote 2a: I think it was probably the fact that the difficulties weren't constantly being medicalized—that the clinician was working with the young person to kind of acknowledge their difficulties and support them and being able to understand why they might be experiencing it. (Practitioner 1001)
Quote 2b: The therapeutic assessment is good at being sort of sensitive enough in a way, but also, more like practical about it. Like, it's not getting too emotional in a way, if that makes sense, about self-harm. But it is sensitive. (Practitioner 1002)
Quote 2c: The [self-harm] cycle. I think it's really useful to kind of to really break down what like self-harm is and to help people kind of really distinguish between those kind of thoughts and feelings and then actions and really like segment them. I think for a lot of people, it was maybe the first time that that that had happened. And for anyone who kind of gets that it's almost like it's a crash course in self-harm and why people do it. It's quite an enlightening moment to be like, oh, so maybe that's why. And just to be able to think and spend a bit of time noticing when these feelings come up, can be really useful for people. Yeah, I think there's been some like quite early on breakthrough moments for young people, and they're like, oh yeah. (Practitioner 2014).
Quote 2d: I think [the self-harm cycle] can help with formulation for when we hand over, because with the therapeutic assessment, I found it much easier to write the formulation afterwards because we had that in depth understanding of the dynamics around the self-harm, like the intrapersonal processes and I used some elements of that in a recent case in my post A&E review as well, where I identified that the young person was feeling very guilty and that was driving the self-harm. So I think it's the added benefit that they get some tools as well, more safety planning tools, some more strategies that they can then bring wherever they go and it can be built upon. By the local CAMHS team, or counselling services or whoever is taking them on after. (Practitioner 1020)
Quote 2e: A lot of the focus around crisis work is on risk assessment and that can be organised around a kind of risk averse type model, whereas therapeutic assessment really looks at wider aspects of factors contributing to self-harm and distress. (Practitioner 2001)
Quote 2f: So just the therapeutic assessment gave an in depth, the base of what we were going to be working on. (Practitioner 3308)

**Table 3:** Quotes for theme 3.

Theme 3: Harnessing young people’s strengths and motivating change
Quote 3a: [The crisis safety plan] doesn't have that extra page - what do you need to feel okay in that? And I think that that page does provide a lot of information that I think young people don't realise they need, but also we as practitioners don't realise they need in the sense of like, you know, when you ask them, what do you feel like? What do you need to feel okay. Like sleep, or chatting to my friend. And suddenly you give importance to these things that they were doing every day, but they didn't realise they were doing. And you gave them a bit of routine to realise that actually they needed to make an active effort to do those things because they know that if that doesn't happen, they won't be feeling okay. (Practitioner 2009)
Quote 3b: We put into the enhanced safety plan instead of cutting because in the session she started using the coloured pens to draw the [self-harm] cycle. She was taking it then scribbling on her arms. And then she identified [as a coping strategy] from that session. Maybe it's best to do this when we're trying to find the, the less the like negative way of coping so in that way. That had helped … And they reported, like, how every week, like things were getting better and better and they were not harming. (Practitioner 3303).
Quote 3c: Sometimes they will tell me, like, ‘Oh, I don't think that works’. Or sometimes people like, ‘Oh yeah, I think that would work’. So they're able to tell you where they agree. So I suppose it's like motivational interviewing, right? You want them to come up with the ways of change and not give it to them- because we don't like being given stuff. (Practitioner 3206)
Quote 3d: I think it works well not being in like active crisis [to do the ESP]. Because I think sitting down with someone in A&E and thinking about what coping skills can you use? I don't think many people would be able to identify what that would be. So I think an opportunity to work with young people a bit longer term post-crisis when things are a bit more calm, to think about what's going to help you here? I used to work in DBT therapy. A lot of that work was about, alright, we're not in crisis now. We have to think of the skills now. That way when we're heightened or dysregulated, we're not panic again, [so they can] think, oh, what can I use? So I think with the enhanced safety plan, that was very similar to that. I think that's even something we could do in seven day follow-ups. So I think, yeah, I think it does work a bit more effectively. (Practitioner 3202)
Quote 3e: I still provide that space to provide that holding containment if they needed to tell me something, or if they felt like they wanted to talk about something, whether it's kind of potential risk concerns. So one of our sessions, they actually came in and spoke about a friendship situation that had been quite upsetting for them. And I found tailoring the structure of the session really helpful in building that relationship in helping the young person feel heard when they had a problem—but also being able to draw out the resources that they've demonstrated in responding and coping with that difficult situation. And then going back to what's been better—does that make sense? Because personal preference—I don't like going straight into the solution focused. I don't want to have them feel like they don't have a space to bring the negatives. But then in allowing that space for negative, you can then transition that to focus on what happened that went well. So I think that helped with the therapeutic relationship and containment. (Practitioner 3206)
Quote 3f: I think the scales are really helpful, because sometimes if you're asking: ‘How can things be better?’. It's a very overwhelming question because actually it might be lots of things, but actually to be like: ‘Okay, what, where's your mood at now?’ And then: ‘How do we get you just one point above?’ I think people found that really helpful rather than because we always ask people, people to rate their mood anyway. It's like CAMHS’ bread and butter asking people to rate their mood. And then often, you know, if it's like: ‘Oh, but how would it be ten out of ten?’ That's, you know, they want the world to change. But actually to look at just one step up makes it really helpful and breaks down a bit. (Practitioner 1002)
Quote 3g: I think like reflecting on each session after like identifying like having them identify what's been better and like rating like, it was nice to see people rate like their idea of better and being closer to it. Even when maybe they didn't like think it was. Like, okay, what did they rate it last time? When they'd say like a number like, oh, that's not that great. I'm like, well, you did say it like it was lower before. And I guess like calling attention to that, it's like you may not think it's like 100% but we are we are higher than we were last time. So I think it's like the own perception of like oh, I'm not doing that great, but it's like but when we look back on progress like it's not as where we were before so. (Practitioner 3202)

**Table 4:** Quotes for theme 4.

Theme 4. Carer involvement leads to better outcomes when personalised but is constrained in practice
*Sub-theme 4.1. There are barriers to carer involvement, as not all young people want their carers involved and carers may not be available or prioritise support for themselves*
Quote 4.1a: But I think with the ones the two that I had were a bit older, their family wasn't really involved and they didn't really want them to be involved in both cases. So I think that's something that happens with age too—like with 14 you kind of have to have them involved. But when they're at that grey area, 16,17, they were kind of at the place where they were able to make decisions without them for the most part. But some things that came out I was like, yeah, I do have to tell your parent about that. But for the most part it wasn't like we have to have your parent involved in every stage. It was like, okay, this is for you, this is your experience, this is your work. How are we going to do that? (Practitioner 3202)
Quote 4.1b: It can be really tricky where the most important thing to do is have that that joint session and the young person being like, ‘no, I don't want’. And you've really got to respect that. And, you can push back, but you know, there are limits. And if you are treating them like an expert in their own life, then you can't be like overriding their decisions. So it could be really tricky. But then when you do and if you are successful with managing to get them in the room together, yeah, something like a solution focused model, more of a relational like systemic model is a really helpful way of having useful conversations and it not descending into like anarchy and chaos and people throwing chairs and stuff. (Practitioner 2014)
Quote 4.1c: [Parents] prioritise the young person. They feel like in that moment, it's not them that needs the support, it's the young person. And any resources that are available should be focused on the young person. And once the young person is in that better position, they then feel like, okay, maybe I can. And it I know it still needs a little bit of encouragement because with a lot of them, it did require several times I had to ask. And they never really said no, they were all like, oh, I'll consider it, I'll have a think about it. They never explicitly said no, I don't need it. (Practitioner 2009)
*Sub-theme 4.2. Individual work with carers gives them their own space to ensure they are supported, which has a positive impact on the young person*
Quote 4.2a: [In usual care] if there's risk, you really want to tell the parents, but it's very much like, it feels quite administrative traditionally where it's like checking in, letting them know kind of about any risk, about anything that the young person wanted you to mention to their parents. Whereas here it's been really helpful working a bit more therapeutically with the parents. It's been really nice to do that. (Practitioner 2014)
Quote 4.2b: It's really, it's really cool that you can focus just on parents and what they want, but you can kind of feel quite confident that that's going to have an impact on like the most important person, which is the young person. (Practitioner 2014)
*Sub-theme 4.3. Joint work between young people and carers led to shared understanding of the difficulties, better communication and recognition of progress*
Quote 4.3a: I think with parents, it's always really helpful. It's when we do our typical safety plans anyway, when we show it to parents afterwards. Well, if the kids want, but usually, and it can be really helpful because a lot of young people don't talk to their parents about how they're feeling and what might cause them and what might make them feel better. And sometimes it can open up a dialog between them, which can be really useful. Like if someone said, actually they just want to be on their own when they feel sad, they need some time. Parents often are doing the opposite because they're like: ‘Oh, you seem sad. What's wrong?’ And they're kind of pestering. So then it can help open a dialog about what you do in those situations. So people have ended up agreeing like: “Oh, actually, I'll be alone for an hour and then I'll come and see you,” or “you can check on me.” Or like, you know, then they can form a bit more of an agreement, which I think can be really nice. (Practitioner 1002)
Quote 4.3b: Sometimes parents might have the best intention, but they're not always, like listening to the child. So I think having the visuals of [the therapeutic assessment is] really helpful. Because they aren't always hearing what the young person's saying, even though they're like trying. They mean well, but they're not hearing it. I think sometimes it can help a parent because quite often parents are very angry about their child self-harming. And I think actually seeing the cycle of it, it almost it explains why they did it. You can see how and why the young person got to that point. Obviously you want to divert away from the self-harm, but you can only see like: ‘Oh yeah, I can see how they end up doing that.’ And then parents can kind of understand a bit more rather than just be angry that that their child has done it, if that makes sense. (Practitioner 1002)
Quote 4.3c: So then it's like the parent was part of the end of the session and they get to give their feedback and a lot of the times, the parents would say some really nice things that the young person maybe never had heard them say before. It was a really nice, sweet moment. (Practitioner 2009)
Quote 4.3d: They almost feel lost on how to support their child like they want to support the child. They don't know how. The more they become involved in our in our sessions and get involved in the conversations, they're picking up pointers and I know i they carry that home also. I remember one parent saying she picked up some of the questioning like, ‘what difference does it make?’. And when her daughter was saying that she'd ask, ‘okay, so what difference will that make?’ She was picking up on so she was picking up [solution focused language] without her knowing she's consciously doing that. And I think that helped the young person to feel that the mum is being more understanding and is listening and is wanting to support. And it's not like the mum wasn't always doing that—it's just changing the phrase. (Practitioner 2009)

**Table 5:** Quotes for theme 5.

Theme 5: SASH worked well for most young people, although may be less suited to those who are less able to express themselves, have more complex difficulties or difficulties that are more outside of their control
Quote 5a: I feel like [SASH] tends to work well with young people who are quite motivated to change. Because I've heard of colleague's examples when they've done SASH and a young person like did not engage well. Or struggled to engage. Maybe it wasn't the right fit—or they sometimes they might really struggle with kind of thinking about what might be different or like better. I think in those circumstances that can be quite a struggle. It just turns out that both my young people I would describe them as very motivated and they're quite chatty in nature. So I think that worked very well for them. (Practitioner 3206)
Quote 5b: Yeah, with the older patients, I think they were more engaged with the intervention. And probably understood things better. As opposed to the younger ones. I worked with 16 year old and a 17 year old. I know I attempted to work with a 13 year old so there was a there was a very big difference with regards to so the young ones would have needed a lot more time to get into it. (Practitioner 3308)
Quote 5c: But a new person or somebody that is a bit new [to the crisis team], that is going through maybe difficulties and has ended up taking an overdose or have harmed herself, either the 1st or the 2nd time or third time. [SASH] is an eye opener to them. They may not even be aware of their core pain, but we help them to actually identify it. I felt like with the right clients, it's very, very good and [SASH] will help them to really change their life or understand things further. (Practitioner 21001)
Quote 5d: When I first got kind of the referral information, and when I kind of met them in the first like 7-day follow-up, I had quite a few concerns. There were quite a few warning signs that are areas of concerns to me. And one big thing was the low self-esteem and I was querying body dysmorphia, because this young person was very fixated on their appearance and their weight. So they were really kind of restricting the food intake and this young person was someone who had experienced ongoing bullying and social difficulties. And there was a history of domestic violence and living in refuges. So that was a quite a big warning sign for me as to whether the SASH intervention would have been sufficient to addressing this. And after the first session, I went in with the idea that, okay, the SASH might be beneficial in thinking about like improving her self-confidence, or like, thinking about how she responds to stressors. But I was thinking initially that I would be referring this young person onwards to longer term support in CAMHS for more specific treatment. And then I think between maybe the 1st and the 2nd solution focused session, there was such a big change in the young person. Yes, I honestly didn't expect [it]. (Practitioner 3206)
Quote 5e: Her best hopes were around connection really with family and being understood and being listened to, and she even expressed to the school at some point. That it's not helping because I obviously care about her, but I'm the wrong person to care about her. She wants her parents to care about her and give her that attention. So we also saw a bit the limits of SASH and the intervention. (Practitioner 1020)
Quote 5f: I think it was helpful to have the structure because for people that are neurodivergent, it can be really helpful to have a clear structure. And often they like to know what we're going to do in each session. And it reduces the anxiety if you can be like, this is what we're going to do. And then they know what to expect for the next session, which is really helpful. (Practitioner 1002)

**Table 6:** Quotes for theme 6.

Theme 6: A paradigm shift for practitioners from focusing on problems to solutions, but over time practitioners gained confidence and motivation to using the SASH approach and continue to use it in their practice
Quote 6a: I remember when people were saying, ‘why we're not exploring more about the problem, about the difficulties, about the struggle?’ and obviously I understand where they're coming from because, you know, we have trained to think this way. (Practitioner 1003)
Quote 6b: Just focusing solely on like what's been better like that, I guess like I said it felt a bit unnatural for me. I validate a lot and, not that I wasn't validating. But I just think it was a shift of like. Validation versus we need to talk about what's been good. So I think like trying to find that balance where I don't seem like I'm just like purely dismissing it. (Practitioner 3202)
Quote 6c: I think naturally you always go into a focus on the self-harm and focus on the negative and focus on the on the difficulty and you go into kind of safety planning and risk aversion mode. And that's how I would always kind of practise as a social worker and as a therapist. So kind of seeing this way of like having conversations with people where you are attending to the risk, but also trying to look difference. I think it really has changed the conversations I have in all kinds of aspects of my role… I think it's hard to imagine kind of going back now to a way of being problem focused. (Practitioner 2014)
Quote 6d: I have heard a criticism from other clinicians, that [SASH intervention] was very repetitive and that was quite tiring or boring even for them to deliver it. And the young people were finding it very repetitive. (Practitioner 1003)
Quote 6e: So, it's like, training yourself to think in a certain way. So, when I repeat things, I'm just trying to direct you in a new way of thinking. Of basically finding solutions, understanding what is that right in yourself, finding the obstacles in your way, and what the solutions would be. And dragging information from what you have done already before that was worked well and was helpful. So, it's about the mindset really. So, I didn't find the repetition necessarily bad because of that, as long as it is relevant. As long the young people find it interesting. (Practitioner 1003)
Quote 6f: Just seeing that young people can take things on board and can learn some coping skills in a short period of time, made me more hopeful. That what we do is effective. To keep them safe. (Practitioner 1020)
Quote 6g: What surprised you about working in a solution focused way…that focus on the difference. I think what surprised me was the way that I felt afterwards, after learning about it and then after delivering it. That was a big surprise because you just feel better basically, compared to working in a CBT session, interpersonal psychotherapy session or something like that. And you just feel different. You feel a bit better. (Practitioner 2014)

Adolescents may present to a range of health care settings with self-harm, including primary care. Emergency Departments (EDs) provide an important opportunity to support young people whose self-harm has resulted in hospital attendance, a group who are at elevated risk of repeat self-harm and suicide. It is estimated that each year in England, 21,000 adolescents aged 12–17 present to hospital EDs following self-harm (10). In England, the National Institute for Health and Care Excellence (NICE) recommends a psychosocial assessment by specialist mental health practitioners in the ED for young people aged up to 18 years who present with self-harm, followed by a 7-day follow-up (11, 12). Practitioners from Child and Adolescent Mental Health Services (CAMHS) crisis teams conduct psychosocial assessments to assess mental health, risks, and needs, and agree on a management plan. Follow-up within 7 days seeks to provide appropriate aftercare, including risk assessment and onward referrals. Depending on need, after discharge from the crisis team, young people may be referred to community CAMHS or specialist services. While NICE recommends a comprehensive psychosocial assessment and follow-up care for all children and young people presenting to hospital following self-harm, there is substantial variation in the extent to which these guidelines are adhered to. Although national data are not available, an audit of two paediatric EDs found that there was no evidence of a psychosocial assessment in the medical records for 34% of children and adolescents presenting to hospital following self-harm (13). An audit found evidence of care and/or safety planning in 68.9% of young people aged 8–17 years (14), and another found that 8% of young people did not receive a 7-day follow-up session (15). While based on small-scale studies, these suggest variation in the delivery and documentation of recommended care for young people presenting to hospital with self-harm.

Emergency departments provide a critical opportunity to support young people following self-harm, but rising demand for emergency, inpatient and community care and fewer resources present challenges for delivering optimal support in this context. EDs have seen growing demand, with paediatric mental health admissions having increased by 65% between 2012 and 2022 in under-18s (16). There is a growing body of evidence in adults that has reported difficult and stigmatizing experiences of help-seeking in EDs and a lack of onward care (17–19). Similar experiences have been reported by young people, with a recent meta-synthesis finding that EDs often exacerbated distress and were unable to meet the needs of young people (20). Young people report that difficult experiences in the ED deter help-seeking and contribute to vicious cycles of repeat ED attendances and escalating suicidality (21). As EDs focus on urgent assessment of self-harm and risk, there is little opportunity for understanding the reasons underlying self-harm in the young person's life and supporting the young person to manage the thoughts and feelings leading to self-harm. A key issue in CAMHS crisis provision is that young people are typically discharged to their GP or community CAMHS team, where they may be placed on a waiting list for further treatment. Median wait times for treatment in CAMHS are 8 months, with some young people waiting over a year, exceeding the UK government target of four weeks (22). Evidence shows that while waiting for mental health treatment, young people's often experience exacerbation of mental health problems and increased risk of crisis presentations (23–26). Brief interventions at the point of crisis may therefore help to improve support for young people in crisis, while also reducing demand on community CAMHS teams.

While studies in adults have demonstrated the feasibility of delivering brief psychological interventions following self-harm presentations to EDs (27), evidence for comparable interventions in adolescents remains limited. Existing evidence suggests that interventions are most likely to be helpful when they actively engage young people, promote an understanding of the factors contributing to self-harm, and support the development of personalised strategies for managing future crises (28).

The Supporting Adolescents with Self-Harm (SASH) intervention was developed to address these needs through a brief, collaborative and strengths-based approach. Drawing on Therapeutic Assessment (TA), Enhanced Safety Planning (ESP) and Solution-Focused Brief Therapy (SFBT), SASH aims to support young people's agency and resource activation following a self-harm crisis. The intervention helps young people develop a shared understanding of the personal, relational and emotional processes associated with their self-harm, identify practical and individualised strategies for maintaining safety, and recognise existing strengths and sources of support to facilitate recovery. Central to the intervention is a collaborative formulation and planning process intended to help young people move from crisis management towards a greater sense of understanding, control and future possibility.

The SASH intervention comprised up to six sessions with young people and their parent(s)/carer(s). The initial session integrated TA (29) and ESP (28). TA involved the collaborative development of a personalised formulation of self-harm, and ESP involved the co-construction of an individualised safety plan using the young person's words and preferences. Subsequent sessions drew on principles of Solution-Focused Brief Therapy (30). SFBT sessions focused on helping young people identify their preferred future, recognise existing strengths and resources, explore occasions when difficulties were less prominent, and develop small, achievable steps towards desired change. Sessions were primarily delivered face-to-face, although remote delivery was offered when preferred by the family. Parent/carer involvement could include joint sessions with the young person and/or up to two individual parent/carer sessions. Full details of the SASH intervention have been described elsewhere (28).

The SASH intervention was underpinned by a logic model that proposes that rapid follow-up care, delivered by trained and supervised practitioners using TA, ESP, and SFBT, will reduce self-harm and improve well-being (see Figure 1). Through collaboratively exploring adolescents' self-harm experiences, identifying points at which the self-harm cycle can be interrupted, building shared understanding between the young person and their carers, improving young person and carer understanding of self-harm, developing personalised safety plans, and building on strengths, resources, and existing coping strategies, the intervention aims to promote continuity of care, strengthen the therapeutic relationship, enhance self-awareness, increase confidence and hope, and foster a greater sense of agency. These mechanisms are expected to support behavioural change, improve communication and young person-carer shared understanding of self-harm, leading to improved well-being, quality of life, and service experiences, and reduced self-harm. Long-term impacts are anticipated for young people, carers, staff, and the wider health system, including fewer emergency presentations and specialist referrals, improved functioning, and reduced costs.

**Figure 1:**
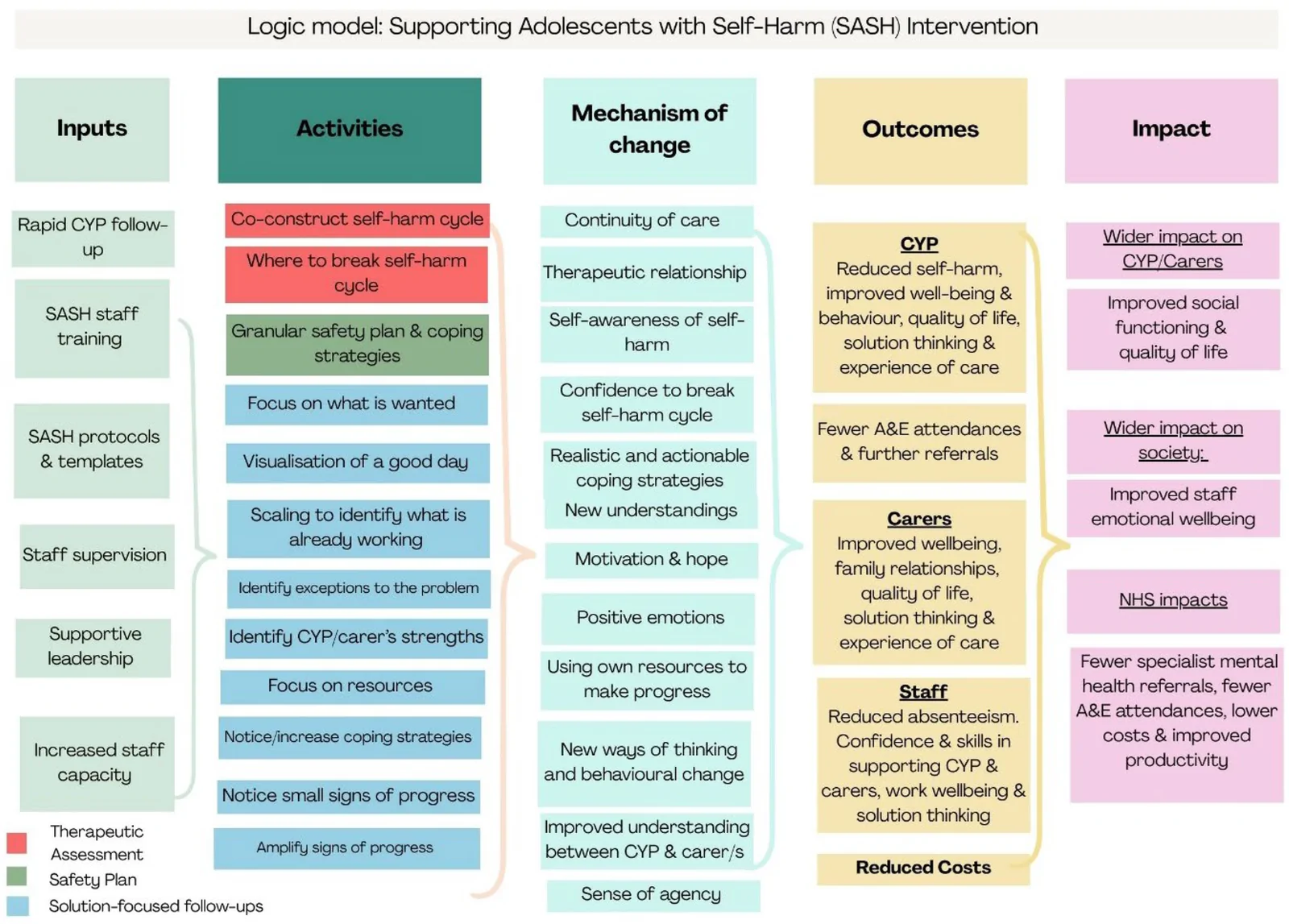
Logic model for the SASH intervention. Reproduced from “SASH intervention logic model” by Rose McCabe, Sally O'Keeffe and Maria Long, licensed under CC BY.

The present study forms part of the SASH trial, which tested this brief intervention for young people presenting to EDs with self-harm or suicidal thoughts accompanied by recent self-harm (31). The intervention was based on the best available evidence of approaches for supporting adolescents experiencing self-harm and/or suicidality (28). Alongside the evaluation of trial outcomes, a qualitative process evaluation was undertaken to explore the experiences of participation and delivery. The present article reports practitioners' experiences of delivering the SASH intervention within the trial.

We addressed the following research questions:

1. What were CAMHS crisis practitioners’ experiences of delivering the SASH intervention, including TA, ESP, and solution-focused follow-up sessions?
2. What are the potential mechanisms of the SASH intervention that may lead to better experiences and outcomes for young people?
3. What were practitioners’ experiences of involving carers in the intervention?
4. Which young people did practitioners feel that SASH worked well and less well for?
5. What impact has the SASH intervention had on practitioners’ wider clinical practice?

## Materials and methods

### Setting for the study

This study was part of the SASH trial (31). The SASH trial was a multicentre, randomized controlled trial to assess the clinical and cost-effectiveness of the SASH intervention in reducing repeat self-harm for adolescents. In standard care (and TAU in the trial), young people presenting to the ED following self-harm receive a psychosocial assessment from mental health practitioners from the CAMHS crisis team that covers that ED, and then for a 7-day follow-up, as specified in NICE guidance (11, 12)—although CAMHS crisis care provision varies considerably in practice (32).

A total of 78 adolescents were randomised to the SASH intervention arm. Of those, 54 (69%) received the SASH intervention, including all three components (TA, ESP, and SFBT). Twenty-three participants (31%) did not receive the SASH intervention for the following reasons: the young person declined the sessions (*n* = 14), risk management/escalation (*n* = 6), improvement that meant further sessions were not deemed necessary (*n* = 2); young person relocated (*n* = 1). Among young people allocated to the SASH intervention arm, 62% of carers/parents were involved in at least one session, either jointly with their child or in an individual session with the practitioner.

A total of 154 adolescents aged 12–18 years were recruited into the trial soon after discharge from the Emergency Department (ED), following presentation with an episode of self-harm or suicidal ideation (with self-harm in the past month). The study took place across three NHS mental health trusts and nine EDs in West, Central, North, and East London. Adolescents recruited into the trial were randomised on a 1:1 basis to the SASH or TAU arm. The SASH intervention, delivered by practitioners in the CAMHS crisis teams, aimed to reduce self-reported self-harm at the 6-month follow-up assessment.

Practitioner participants were NHS mental health practitioners from varied professional backgrounds working in or allied with CAMHS crisis/urgent care teams delivering follow-up care to young people after presenting to ED with self-harm (e.g., mental health nurses, social workers and assistant psychologists). Training consisted of 1.5–3 days delivered in-person or online. Assessment of the fidelity of the intervention delivery was carried out and will be reported as part of the trial outcome paper.

To ensure complete and transparent reporting, we were guided by the COnsolidated criteria for REporting Qualitative research (COREQ; see Supplementary Data Sheet 1) (33).

### Data collection

A qualitative process evaluation was conducted as part of the SASH trial to explore the experiences of young people, carers, and practitioners participating in the trial (31). Practitioners were invited to take part in qualitative interviews at the end of their involvement in the trial. All practitioners who had delivered the SASH intervention to at least one young person were invited to take part, although three had left by the time the interviews took place. In addition, senior clinicians from the participating CAMHS crisis teams were invited to be interviewed.

Practitioners had provided written consent at the outset of their involvement in the trial, including for participation in a qualitative interview. They were invited to take part in the interview via email, and if they agreed, the interview was scheduled to take place over Microsoft Teams. Each interview was facilitated by a member of the SASH team, all of whom were postgraduate/postdoctoral researchers in the field of health services research, with training in qualitative interviewing. The team included both male and female researchers. Several members of the research team were involved in the intervention training and supervision, so they had prior relationships with practitioner participants at the time the interviews took place. Only the interviewer and participant were present in the interviews. Interviews were guided by a topic guide, which was used flexibly (see Supplementary Data Sheet 2). The topic guide explored practitioners’ experiences of delivering the SASH intervention, including TA, ESP and SFBT follow-up sessions, and the impact it had on their practice and professional development. Interviews took place between January and October 2025. They were video-recorded via Microsoft Teams and automatically transcribed, and the transcripts were then fully revised to produce verbatim transcripts. Any identifiable information was removed. The length of interviews ranged from 19 to 95 min (M = 46.26). Field notes were written after each interview by the interviewer. These recorded contextual information about the interview setting, reflections, and preliminary insights to inform the analysis.

### Participants

Of the 23 practitioners who delivered the SASH intervention in the RCT, 15 (65%) took part in a qualitative interview. These 15 practitioners delivered the SASH intervention to between 1 and 7 young people each, with the average being three young people per practitioner. In addition, four senior members of staff who acted as local Principal Investigators in the study sites were interviewed. Hence, 19 clinical members of staff from across the three participating mental health Trusts were interviewed. Participants had an average age of 36.29 years (SD = 10.91) and 79% were female. Their professions were nurses (*n* = 8), social workers (*n* = 4), psychiatrists (*n* = 3), assistant psychologists (*n* = 2), occupational therapist (*n* = 1), and support worker (*n* = 1).

### Data analysis

Data were analysed using thematic analysis (34) in NVivo 14.0 (35). An inductive approach was used. Line-by-line coding was carried out by the first author for all 19 interviews. A total of 549 line-by-line codes were created. The line-by-line codes were then grouped into 38 initial categories, which were then analysed by the first author to develop them into conceptual themes/sub-themes. These were reviewed with the rest of the team to identify overarching themes that addressed the research questions.

The researchers involved in the analysis worked on the SASH trial and were involved in developing the intervention and the training and supervision of practitioners. As such, we were aware that our alignment with the principles of the SASH intervention could influence data interpretation. Throughout the analysis, we aimed to remain grounded in participants’ accounts by returning repeatedly to the original data and ensuring that interpretations were supported by participants’ narratives. We actively attended to both positive and negative experiences of the intervention, with the aim of capturing the complexity and diversity of practitioners’ experiences. Regular team meetings encouraged critical reflection on our interpretations.

## Results

Six overarching themes were identified. Figure 2 shows a thematic map reflecting how the themes fit together.

**Figure 2:**
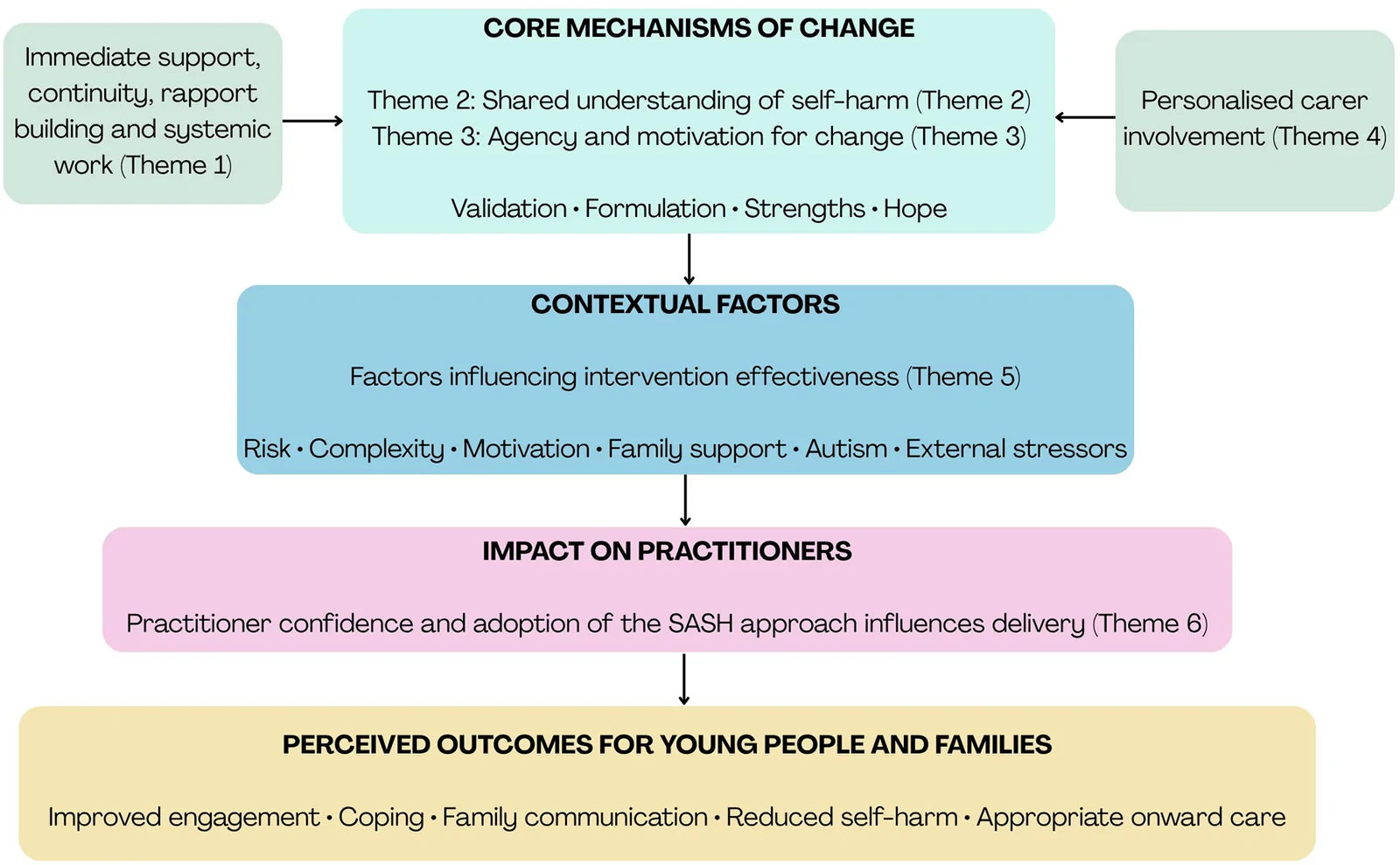
Thematic map to show how the themes fit together.

### Theme 1: A transformation in practice, bridging the gap in crisis care by offering an immediate therapeutic intervention and time to build rapport and work with the system around young people

The SASH intervention was perceived by practitioners as a transformation in practice, enabling them to offer an immediate therapeutic intervention to young people. Being able to offer continuity of care over a number of follow-up sessions was seen as beneficial for young people, offering immediate intervention and bridging the gap in crisis care, where young people are typically discharged to their GP or placed on a lengthy waiting list for treatment in community CAMHS (Table 1; Quote 1a). This continuity meant young people did not have to repeat their story to a new practitioner.

The extended time they had to work with young people meant they had longer to work with the system around the young person, including their carers, school and in some cases, social services (Table 1; Quote 1b). This enabled them to offer holistic care to the young person that was tailored to their individual needs. Practitioners often described the time it took to build a relationship with young people and to engage them in the intervention (Table 1; Quote 1c). Practitioners acknowledged the importance of the flexibility of the model, which meant they could pace the sessions according to the young person's needs. For instance, sometimes they extended the TA or ESP into the second session, so as not to rush the process.

Practitioners felt this brief intervention was enough for some young people to be discharged to their GP, who ordinarily might have been referred to CAMHS for further support and would have waited for many months for treatment. One practitioner suggested that even where further intervention was required post-SASH, the SASH intervention meant they had more time to understand the young person and their needs, leading to better-informed referrals through truly understanding their needs, and ensuring the most appropriate onward care (Table 1; Quote 1d). For some lower-risk cases, the SASH intervention meant they received more support than they would ordinarily receive, which practitioners believed was likely to contribute to a better prognosis, including preventing further A&E attendances. Thus, regardless of the severity of the young person's presenting difficulties, having this extended opportunity to work with them provided a safety net to support young people through crisis.

The SASH intervention was perceived by practitioners as potentially preferable for some young people who might have been overwhelmed by more intensive therapy, suggesting a brief intervention may be a beneficial first-line treatment for young people following a crisis presentation (Table 1; Quote 1e). Immediate support and continuity were regarded as best practice, and practitioners believed these would lead to the best outcomes for young people (Table 1; Quote 1f).

### Theme 2: Patient-centred care that builds shared understanding between the young person, carer and practitioner

Practitioners emphasised how SASH focused on validating, acknowledging and understanding the young person's difficulties, rather than medicalising them (Table 2; Quote 2a). Practitioners reported how the approach was collaborative, involving young people in every aspect of the intervention, which placed them as the expert in their own experiences.

The TA was viewed by practitioners as instrumental in helping the young person to better understand their self-harm through co-constructing the self-harm cycle, which provided a powerful visualisation of the factors contributing to their self-harm. This process was seen as an in-depth, yet practical and sensitive way of exploring the young person's self-harm (Table 2; Quote 2b). This was seen as helping young people to see how their personal feelings, thoughts and behaviours linked together to contribute to their self-harm and make sense of the difficulties they were experiencing, which was “enlightening” in helping young people to distinguish between the thoughts and feelings that contributed to their self-harm (Table 2; Quote 2c). One practitioner described how young people often feel guilty or stressed about their self-harm, but the self-harm cycle seemed to help validate and normalise their self-harm and start to find solutions to break the cycle.

Having co-constructed the self-harm cycle, practitioners described how, in turn, this helped them to better understand the young person's self-harm and contributed to their risk assessment and formulation (Table 2; Quote 2d). For example, it helped them to better understand the impulsivity of the young person's self-harm and to fully explore the factors contributing to their distress (Table 2; Quote 2e). The understanding provided by the TA provided the foundation for understanding what they would work on in subsequent sessions (Table 2; Quote 2f). This shared understanding extended to carers, with whom the self-harm cycle was sometimes used to help them to understand their child's self-harm (see Sub-theme 4.3).

### Theme 3: Harnessing young people’s strengths and motivating change

A strand that ran through all three components of the SASH intervention was the resources- and strengths-based approach, which enabled practitioners to support young people in finding their own coping strategies, strengths and solutions, thereby motivating change. Practitioners felt this promoted agency and independence by helping young people recognise their capacity to manage difficulties and gain confidence to make changes in their lives.

The strengths-based approach begins in the TA, where practitioners described how, having co-constructed the self-harm cycle, young people were often able to easily recognise where they could break the cycle. This focus on strengths is also incorporated in the ESP. The first section of the ESP focuses on the young person when they are well and was well received by practitioners – as this is something that is not included in their usual safety plans. They spoke about how this was a positive way to start the ESP, drawing out their values and resilience, and helping them to recognise the everyday things they could do to improve their mood (Table 3; Quote 3a). While some practitioners noted that it took a lot of prompting for some young people to identify the positive things in their lives, this approach was seen as more comprehensive than their usual safety planning approach, providing a helpful reminder of the things young people could do to make themselves feel better.

Practitioners described how the second section of the ESP—which focuses on coping strategies—then enabled them to work with young people to identify a range of positive ways in which they could cope with and release their mental pain. Practitioners liked that the ESP was broken down into small categories to help young people find a range of coping strategies (e.g., distractions, environment, trusted people, and professionals), and this was seen as providing greater depth than their usual safety plan, which focused on restricting the young person's access to means for self-harm and calling crisis helplines. The coping strategies were seen as truly personalised; for example, one practitioner described how a young person identified an alternative way of coping with self-harm, which was to draw on her arms instead, which she then used enabling her to stop self-harming (Table 3; Quote 3b). Practitioners described how the ESP included sections to explore what might stop the young person from using their coping strategies and steps they could take to overcoming these barriers, all of which made it more in depth their usual safety plan, maximising their chances of using their coping strategies. Practitioners also emphasised the importance of getting the young person to generate their own coping strategies, so they were personalised to the individual and what they felt would be helpful, rather than telling them what to do. One practitioner compared this process to motivational interviewing, where you want young people to come up with the ways they can change for themselves, to motivate change (Table 3; Quote 3c).

Having the opportunity to complete this ESP after the initial crisis, when the young person was not in a heightened state of distress, was seen as helpful in enabling the young person to reflect on coping strategies and what might be helpful (Table 3; Quote 3d). This was seen as distinct from the usual safety plan which is focused on practical measures that will keep the young person safe (e.g., restricting means for self-harm) whereas the ESP was seen as a more person-centred approach that allows the young person to reflect on their strengths and resilience. The drawback of the ESP was that some practitioners questioned whether young people refer to it in times of crisis, thus questioning how useful they find it.

The strengths-based approach is the main feature of the solution-focused follow-up sessions. This was seen as distinct from their usual practice in which they focus on the young person's problems. While practitioners often had reservations initially about working in a solution focused way, for fear of not allowing space for young people to speak about their problems, in practice, practitioners described how they found they were able to find balance between allowing space for problems but then using this to draw out the strengths young people had displayed in coping with their difficulties (Table 3; Quote 3e). Practitioners described how the solution-focused approach provided a different way of having conversations with young people and carers, which was a refreshing change for young people who may have had to repeat their story to many healthcare professionals in the past, shifting the focus to a focus on the future. This led to more positive interactions compared with the risk-focused conversations practitioners were typically used to having with young people in this context.

Practitioners spoke positively about using scales—which ask young people about where they are on a scale of 1–10, with 10 meaning their best hope has been realised—and described how this approach often motivated young people to find ways of increasing their scores on the scales. Breaking down change young people could make in a granular way was perceived by practitioners as identifying realistic goals for young people (Table 3; Quote 3f). Practitioners reported that they often noticed positive change between sessions, and working in this way enabled practitioners to draw attention to the small steps of progress young people had made (Table 3; Quote 3g). Practitioners often reported that young people enjoyed thinking about the tomorrow questions and the scales, which really allowed them to have deeper conversations that they would usually have, such as exploring why their scores were not lower than they were.

Overall, practitioners described liking the focus on solutions, with one comparing this approach to taking “paracetamol to ease your headache” (Practitioner 1003), with this future-focused talk giving young people a break from their difficulties.

### Theme 4: Carer involvement leads to better outcomes when personalised but can be constrained in practice

Carer involvement was seen as crucial for ensuring the best outcomes for young people, including fostering shared understanding between young people and their carers about their difficulties and improved communication within the family. Where carers were not involved, practitioners described it as negatively affecting the resources the young person was able to draw on. Practitioner experiences of carer involvement are captured in three sub-themes:

#### Sub-theme 4.1. There are barriers to carer involvement, as not all young people want their carers involved and carers may not be available or prioritise support for themselves

While carer involvement was considered important in the SASH intervention, practitioners acknowledged that this was not always possible. Practitioners described how not all young people wanted their carers involved in the sessions, so unless it was deemed essential for the management of risk issues, practitioners would respect their wishes regarding if, how and when to involve their carer(s) (Table 4; Quote 4.1a). In some cases, practitioners found it helpful to initially build a relationship with the young person before bringing the carer in. Practitioners acknowledged that it was not always helpful to have carers involved, particularly if there was a difficult dynamic between the young person and their carer(s), as this could impact their engagement in the sessions. This reflects the need for a flexible approach to carer involvement that maximises involvement of carers, where appropriate and acceptable to the young person, treating them as the expert and negotiating carer involvement with them (Table 4; Quote 4.1b).

In addition to the question of whether the young person wished their carer(s) to be involved, practical issues regarding carer availability for sessions were frequently reported by practitioners. Carers’ working hours often limited their availability for sessions. Practitioners described being flexible to support carers’ attendance or offering sessions over the phone to fit in with their availability, although some felt limited in how much support they could offer over the phone. Practitioners also reflected that carers were sometimes reluctant to take up the offer of support as their priority was to obtain support for their child, but carers were sometimes more willing to accept the offer of support once their child was coping better and had navigated the crisis that had led them to A&E (Table 4; Quote 4.1c).

#### Sub-theme 4.2. Individual work with carers gives them their own space to ensure they are supported, which has a positive impact on the young person

Practitioners reflected that the offer of individual sessions for carers gave space for more therapeutic work compared with usual practice, in which carer involvement is focused on managing risk (Table 4; Quote 4.2a). Practitioners recognised that carers were often struggling as much as the young person, and that the offer of individual sessions provided a containing space for carers. It gave the practitioner time to hold in mind what was going on for the carers, to ensure their needs were met as well. For example, one practitioner described signposting a carer to obtain further support, so that she received the help she needed. Practitioners reported feeling they connected well with carers who took up the offer of individual sessions, and it was rewarding to see carers find the sessions helpful.

Practitioners described a range of ways in which these individual sessions were used, depending on the needs of the family. Practitioners often reported the need to give carers space to discuss the difficulties before drawing on the solution-focused approach. Others offered psychoeducation to help carers better understand self-harm and mental health, which made them able to talk about self-harm in a more empathetic way. Ultimately, practitioners reported finding it helpful to be able to provide some individual support for carers, knowing that it would have a positive impact on the young person (Table 4; Quote 4.2b).

#### Sub-theme 4.3. Joint work between young people and carers led to shared understanding of the difficulties, better communication and recognition of progress

Carers were often included in the ESP, which was seen as valuable for offering helpful ideas for how the young person could overcome barriers to using their coping strategies, and to help carers to understand how they could best support their child in difficult moments (Table 4; Quote 4.3a). This aligned with their usual ways of working with carers, but the joint work went beyond this. Practitioners reported using joint sessions to facilitate collaborative conversations between young people and their carer(s), in which both could express their needs and feel heard. These conversations helped to build a shared understanding between the carer and young person of the young person's difficulties. The self-harm cycle was seen as helpful for enabling carers to better understand their child's self-harm, which in turn led to young people feeling better understood by their carers (Table 4; Quote 4.3b).

Joint sessions were also perceived by practitioners as leading to improved communication between young people and their carers. In particular, the solution-focused approach was seen as a way of helping carers find the language to talk to their child in a non-judgemental way. Practitioners reflected how in the joint sessions, carers would notice what was better and recognise the hard work being done by the young person. It was seen as transformative for young people in having their strengths and progress acknowledged by their carers, which were often things their carers hadn't said to them (Table 4; Quote 4.3c). Practitioners also described how some carers started using the solution-focused language themselves, such as asking their child “what difference would that make?.” Thus, carers picked up techniques that they were then able to carry on at home (Table 4; Quote 4.3d).

### Theme 5: SASH worked well for most young people, although may be less suited to those who are less able to express themselves, have complex difficulties or difficulties that are outside of their control

The SASH intervention was regarded by practitioners as suitable for most young people in the SASH trial. Practitioners reported that young people generally engaged well in the SASH intervention, especially those who were motivated to change and able to express their emotions (Table 5; Quote 5a). Practitioners generally felt that the intervention was suited to young people across the 11–18-year age range for SASH. While practitioners acknowledged some older adolescents were not interested and did not engage in SASH, others engaged well and made good use of the sessions. Practitioners reported that it could take longer to build rapport with some younger people (e.g., 12-year-old), and they needed to take more time to explain things to them (Table 5; Quote 5b).

Practitioners expressed that they felt SASH generally worked best with lower-risk and less complex cases, as well as those who had recently started self-harming, who tended to engage well, and for whom it could be “eye opening” (Table 5; Quote 5c). For those young people who disengaged from the intervention, practitioners understood this as being the result of not having family support to engage, or from their struggle to talk about their emotions and found it overwhelming. However, some practitioners described how those who struggled to talk about their self-harm and emotions finding the solution-focused prompts easier to respond to than the TA and ESP.

SASH was generally seen as less appropriate for young people with longer histories of self-harm and who were well known to services, who might have found that it did not meet their needs. Practitioners suggested that these young people often have external factors that impede the intervention, such as a lack of social support, which makes engagement difficult. Practitioners suggested that these young people might need more intensive interventions to meet their complex needs. However, some practitioners reflected that although they had initially considered some young people too complex for SASH, they observed a drastic improvement in the young person following the SASH intervention, challenging their preconceptions about which cases were suitable for SASH (Table 5; Quote 5d).

Practitioners also raised other issues that impacted the helpfulness of the SASH intervention. Practitioners reported that the solution-focused approach could work less well for young people whose difficulties were less within their control, for example, external factors such as family problems or being bullied. For example, one young person described wanting attention and care from her carers (Table 5; Quote 5e). Practitioners expressed mixed views regarding the suitability of SASH for autistic young people. Some practitioners described it as helpful to have a structured approach that helped reduce their anxiety (Table 5; Quote 5f). Some practitioners, however, reported that SASH could be difficult to deliver to autistic young people, especially if they struggled to identify and discuss their feelings, which could make the TA and ESP challenging. One practitioner described how it worked with some autistic young people and not others, so it isn't “one-size-fits-all” (Practitioner 3202). This practitioner reflected on how surprised she was by how well it worked with one autistic young person, and while it was challenging to work through the ESP with them, they stopped self-harming after this, suggesting it had been helpful for the young person.

### Theme 6: Paradigm shift for practitioners from focusing on problems to solutions, but over time practitioners gained confidence and motivation in using the SASH approach and continue to use it in their practice

The SASH intervention was perceived to be a paradigm shift: the strengths-based approach was initially seen as potentially conflicting with practitioners’ usual way of working, which focuses on risk management. Some practitioners reported feeling concerned or frustrated about not focusing on problems and difficulties (Table 6; Quote 6a). This felt challenging for some practitioners to integrate this approach into their work with young people. For example, one practitioner described initial concerns that the solution-focused approach could be experienced by young people as invalidating (Table 6; Quote 6b). Over time, practitioners gained confidence in validating young people while also drawing out their strengths, and this felt more comfortable for them by starting the session by explaining its objectives so that the young person knew what to expect.

Despite these initial challenges and reservations about the SASH intervention, practitioners described how—with practice—they gained confidence in using it, and it became “second nature” to deliver (Practitioner 1002). Practitioners recognised the advantages of working in this way, such as asking different questions than usual meant they didn't feel like a “broken record” (Practitioner 3107). Practitioners described the focus on young people's strengths, character and personality, and the positive aspects of their lives, positioning them as the experts of their own lives, rather than focusing on psychopathology and diagnoses, as a refreshing change in practice. Practitioners felt more confident over time in attending to risk as they adopted a solution-focused approach (Table 6; Quote 6c).

However, some practitioners reported that the structure of the SASH intervention could feel repetitive to deliver, and worried that some young people might find it repetitive too (Table 6; Quote 6d). For instance, one practitioner described a young person who gave the same responses to the questions each session, and the practitioner questioned whether they were failing to engage the young person effectively. In contrast, other practitioners felt the repetitive nature of the solution-focused sessions could be a productive way of noticing change (Table 6; Quote 6e).

Most practitioners reported enjoying delivering the SASH intervention and noted a range of positive impacts on their roles and professional development. One consultant suggested that practitioners with a psychological background took to SASH more easily. However, the structured approach was perceived as helpful in enabling less experienced practitioners to learn to deliver the SASH intervention too. Practitioners described feeling more optimistic and hopeful when using the SASH intervention, noting that they could offer a brief intervention that is perceived as helpful for young people (Table 6; Quote 6f). Practitioners often reported feeling better when working in this way compared with their usual clinical work (Table 6; Quote 6g).

Despite the initial concerns and challenges regarding the use of the SASH intervention, practitioners described enjoying delivering it and felt ownership of the approach. Most practitioners reported continuing to use aspects of the SASH intervention in their practice. Some practitioners described using the TA, including focusing on triggers, core pain, and the self-harm cycle in their clinical work. Several described continuing to use the ESP, and one practitioner stated that members of their team who had not been involved in SASH had adopted it, suggesting this approach was preferred to their usual practice. Practitioners described a range of solution-focused techniques that they continued to use, including scales, best hopes and miracle questions, and focusing on young people's resources. Practitioners reported feeling increasingly motivated to use these new clinical skills after noticing that young people who were receiving the SASH intervention seemed to be doing well.

Overall, practitioners described valuing the opportunity to learn to use the SASH intervention and gain experience in delivering it, which impacted positively on their clinical skills and professional development.

## Discussion

We investigated practitioners’ experiences of delivering the SASH rapid brief psychological intervention to young people following an ED presentation for self-harm. The SASH intervention provided continuity of care to young people following a crisis, which was perceived by practitioners as a transformation in practice, enabling them to build a shared understanding of the reasons underlying the young person's self-harm, work with the family and system around the young person, and harness the young person's strengths. In doing so, the SASH intervention introduced a therapeutic function into a part of the pathway that is typically focused on risk assessment and crisis management. It focused on collaboration with the young person as an expert on their own life, avoiding the medicalisation of difficulties. The strengths-based approach was regarded as a refreshing shift in practice for practitioners, allowing them to offer a therapeutic intervention immediately following the crisis, thus bridging the gap in crisis care for young people. The SASH intervention may act as an early intervention for young people, supporting them in improving insight or understanding, helping young people identify patterns before they become entrenched and prevent progression to more chronic or severe presentations or self-harm. Provision of this brief intervention was perceived by practitioners as providing enough support for some young people to be discharged to primary care, who ordinarily might have been referred to CAMHS for further support and ended up on a waiting list for treatment.

### Situating the findings in the literature

The SASH intervention was perceived by practitioners as providing immediate intervention and continuity of care to young people, which may substantially address the negative experiences of ED care for self-harm reported previously (20, 36). Practitioners perceived SASH as providing a more therapeutic and responsive model of care by ensuring rapid follow-up in the aftermath of a crisis. This continuity of care is particularly important given that adolescents have a significantly increased risk of repeat self-harm and suicide following discharge from hospital (37).

Practitioners also highlighted the value of offering support during the period between crisis presentation and longer-term treatment. Long waits for mental health care have been associated with increased psychological distress, maladaptive coping, lower engagement, and poorer clinical outcomes (26, 38–40). Practitioners felt that working with young people after the immediate crisis had subsided enabled more meaningful formulation and safety planning than is typically possible in emergency settings. Rather than focusing primarily on risk management and means restriction, practitioners described developing personalised coping plans grounded in the young person's own strengths, resources, and support networks.

Consistent with previous studies of TA, practitioners reported that collaborative exploration of self-harm helped young people understand the links between thoughts, emotions, and behaviours (28). Improved understanding was perceived to facilitate more appropriate onward referrals when further intervention was required. Practitioners also described how co-constructing self-harm cycles helped validate and normalise young people's experiences, potentially reducing self-stigma and enhancing engagement with support, consistent with previous findings regarding TA improving subsequent treatment engagement (41).

A core feature of SASH was supporting young people to identify their own strengths, resources, and solutions. Across the TA, ESP, and SFBT sessions, practitioners reported that young people were encouraged to identify opportunities for change, generate coping strategies, and identify small steps they could make to move towards their preferred future. Practitioners recognised the importance of young people generating their own ways of breaking the cycle, coping strategies and solutions, to maximise the chance of them making use of them. Given young people's developmental need for autonomy (42), this approach may be particularly well suited to this population, empowering them to identify their own coping strategies and focus on what is important to them, and thus enhancing motivation, potentially leading to greater behavioural follow-through (43).

Carer involvement was viewed as another important component of the intervention and is consistent with evidence highlighting the role of caregivers in effective interventions for self-harm (44). Although some young people preferred not to have their carers involved, as cited in previous studies (45), practitioners described benefits when carers were able to participate. Flexible involvement enabled practitioners to provide support and psychoeducation to carers, facilitated collaborative problem-solving, and improved family communication. Practitioners felt that solution-focused conversations helped carers discuss self-harm using less judgemental language and recognise existing strengths within the family. This is important given the communication difficulties frequently reported in families affected by self-harm (46).

Practitioners identified several challenges in delivering the intervention, particularly the solution-focused sessions. For many, this represented a significant shift from the risk-oriented approach that characterises crisis work (19). Some practitioners initially worried that focusing on strengths and preferred futures shortly after a mental health crisis might feel repetitive or invalidating. However, with experience and supervision, they described increasing confidence in balancing the validation of distress with strengths-based conversations. Many reported that adopting this approach improved their optimism and satisfaction in their clinical work. This finding is particularly relevant given the burnout and workforce turnover reported within crisis CAMHS (47), suggesting that strengths-based approaches may benefit both service users and practitioners. The SASH intervention may have wider implications for workforce development, potentially contributing to more positive and sustainable ways of working in high-pressure clinical environments.

Although practitioners generally reported positive engagement with SASH, views differed regarding its suitability for more complex presentations. Some felt the intervention was best suited to young people with lower levels of risk and complexity, particularly those motivated to engage and able to reflect on their experiences. Others described positive outcomes among young people with more complex difficulties whom they had initially expected would not benefit from the intervention. Similarly, practitioners reported mixed experiences when working with autistic young people. These findings suggest that further research is needed to understand which young people benefit most from the intervention and whether adaptations are required for specific populations.

The findings align with principles of personalised and integrated care. Practitioners described how SASH provided timely intervention following crisis presentation, continuity of care, collaborative formulation, involvement of carers, and a strengths-based focus on young people's existing resources. These components are consistent with models of person-centred care, which emphasise coordinated support, shared decision-making, personalised care planning, and recognition of individual strengths and preferences. Framed in this way, SASH represents a shift away from a risk-focused response to self-harm towards a more holistic and collaborative model of care.

The findings may also be interpreted through the Capability, Opportunity, Motivation-Behaviour (COM-B) model, which proposes that behaviour change is influenced by the interaction of capability, opportunity and motivation (48). Practitioners' accounts suggested that SASH may enhance psychological capability by helping young people develop a shared understanding of the factors contributing to self-harm and to identify personalised coping strategies. The SASH intervention also appeared to create opportunities for change by involving carers/parents and wider support systems. Finally, the collaborative and strengths-based approach was perceived to support motivation by enhancing young people's agency, hope, and confidence in their ability to make changes, while also increasing practitioners' confidence in delivering the intervention. These findings suggest that SASH may influence several mechanisms that facilitate behaviour change following self-harm in young people.

### Clinical implications

The findings suggest that SASH offers a feasible model of post-crisis support that can be integrated within routine CAMHS crisis services. Most practitioners reported continuing to use components of the intervention in their everyday clinical practice, indicating that the approach was acceptable and transferable beyond the trial context. Notably, the TA and ESP were perceived as achievable within the 7-day follow-up window, despite pressures on crisis services.

Practitioners regarded the ESP as an important enhancement to standard safety planning because it was developed after the immediate crisis, when young people were better able to identify meaningful coping strategies and barriers to using them. The intervention also encouraged young people to recognise strengths, supports, and signs of well-being, potentially increasing the likelihood that strategies would be used during future crises.

The findings also suggest that SASH may be most effective when embedded within a broader care pathway rather than delivered as a stand-alone intervention. For some young people, particularly those with more complex or enduring difficulties, a brief intervention alone may be insufficient. In such cases, SASH may function as an initial engagement and stabilisation phase while facilitating access to more intensive support where required.

### Future directions for research

Future research should establish the effectiveness of SASH in improving clinical outcomes, including reductions in repeat self-harm and service utilisation. Qualitative research involving young people and carers will be particularly important in determining whether the intervention is experienced as helpful and identifying the mechanisms that contribute to positive outcomes and is currently underway.

Although developed for young people presenting with self-harm, the intervention targets broader transdiagnostic processes, including emotional understanding, agency, coping, and strengths-based change. The intervention may therefore have wider applicability for young people experiencing anxiety, depression, trauma-related difficulties, or other forms of emotional distress. Given the increasing demand for CAMHS, future research should explore whether SASH could function as a brief first-line or bridging intervention in settings beyond specialist CAMHS.

Further work is also needed to evaluate the suitability of SASH for neurodiversity or neurodevelopmental disorders more broadly, e.g. autistic young people or those with learning difficulties. Practitioners reported both benefits and challenges, suggesting that adaptations may be required to improve accessibility. Potential adaptations include greater use of visual supports, more concrete language, structured response options, alternative communication methods, and flexible pacing, similar to adaptations proposed for safety-planning interventions with autistic populations (49). Future studies should investigate how the intervention can be tailored to meet the needs of autistic young people and those with more complex presentations.

### Strengths and limitations

A strength of this study is the inclusion of a relatively large and professionally diverse sample of CAMHS practitioners across three NHS Trusts. Interviews provided rich accounts of practitioners’ experiences and included perspectives from nurses, psychiatrists, occupational therapists, social workers, and assistant psychologists. The findings suggest that SASH can be delivered by practitioners from a range of professional backgrounds without requiring highly specialised expertise.

Several limitations should also be acknowledged. We had limited available information about practitioners who chose not to be interviewed, so it is unknown how representative this study is of the wider pool of practitioners who delivered the SASH intervention. Practitioners who participated may have held more favourable views of the intervention than those who declined participation. In addition, the effectiveness of SASH cannot yet be determined because outcome data from the randomised controlled trial are not yet available. Another limitation is that this study does not provide insights from the perspectives of those who received the SASH intervention. Analysis of young people's and carers’ experiences is ongoing and will provide important perspectives on the acceptability and perceived impact of the intervention. Finally, the resourcing of the SASH intervention was not fully explored in the interviews therefore, further work is required to determine the resourcing and economic impact of implementing SASH on a wider scale. Nevertheless, this study offers valuable insights into practitioners’ experiences of implementing SASH within routine clinical settings.

## Conclusion

Practitioners perceived SASH as a promising model of post-crisis care for young people presenting to the ED following self-harm. By providing rapid follow-up, collaborative formulation, strengths-based support, and opportunities for family involvement, the intervention was viewed as addressing important limitations of existing crisis pathways. While implementation would require investment in training, supervision, and service configuration, SASH has the potential to strengthen continuity of care and provide timely support following self-harm crises. Further research is required to establish its effectiveness and refine delivery across diverse populations and settings.

## Data Availability

The datasets presented in this article are not readily available because they contain sensitive and identifiable information. Anonymised transcripts can be made available on reasonable request to the corresponding author. Requests to access the datasets should be directed to sally.o'keeffe@newcastle.ac.uk.
