# Supplementary material for: Supporting adolescents with self-harm: practitioners’ experiences of delivering a rapid brief psychological intervention to adolescents presenting to Emergency Departments
Source: Front Child Adolesc Psychiatry. 2026 Oct 14;5:1959051. doi: 10.3389/frcha.2026.1959051 (PMC13713662; doi:10.3389/frcha.2026.1959051)
Supplement: Supplementary file 2 [file Datasheet2.pdf]

Supporting Adolescents with Self-Harm (SASH)

Interview schedule: Practitioner interview

- Introduce self
- Aim of the interview: feedback, explore of the delivering the intervention and being involved in such
- Confidentiality,
- Stress the following points:
  - We are interested in what is important to you
  - There are no right or wrong answers, it is your perspective that we are interested in
  - You are not obliged to answer; if you don't want to talk about a subject, please say so and will move on

Prompts to check practitioner is happy to explore further sensitive issues: *Is it alright if we talk about that?*

I would like to ask you about your experience of delivering the intervention.

Experience of delivering SASH

1. What did you think of the SASH approach?
   a. How does it compare to usual care (7dfu)?
   b. How did you find delivering SASH to different aged YP?
   c. Did you deliver SASH to any neurodivergent YP? How did that go?
   (Probe: rapid follow-up, session length, number of sessions, and intervals between, order of the intervention elements (e.g.TA first)
      i. If yes, how did the delivery go with this group?
      ii. What adaptations did you make, if any?

2. How did you find doing the therapeutic assessment of self-harm? / 7-day follow-up?
   a. What worked well / not so well?
   b. How did you feel YP and parents engaged with therapeutic assessment?
   (Probe: How did you find: 1) Drawing out the cycle with the YP, 2) Supporting YP to identify feelings, thoughts, behaviours, 3) Supporting YP to identify where to break the cycle, 4) co-producing diagram/being non-directive)

3. How did you find working with YP to develop an enhanced safety plan?
   a. What worked well / not so well?
   (Probe: How did parents engage with the safety plan? How did you find the two different sections?)

4. How did you find delivering the solution focused follow up sessions?
   a. What surprised you about working in a solution-focused way, if anything?
   b. What worked well / not so well?
   c. How did you find using the scripts and handouts? How useful (or not) were they?
   d. Solution-focused is a very different way of working. What do you think of the model?
      i. What do you like / dislike?
      ii. How do you think the YP/parents found it?
      iii. What do you think the benefits are? If any?
      iv. What do you think the drawbacks are? If any?

   e. How do you think this kind of work could be embedded in usual crisis care?
   f. Are there elements that you've taken into your crisis work?

5. How did you find working with YP and their carers together?

   a. What worked well / not so well?
   b. Probe: solution-focused working together

6. How did you find working with parents/cares independently?
   a. What worked well / not so well?
   b. How did working with parents/carers independently impact the work with the YP – if at all?
   c. How did working with parents/carers independently impact the relationship between the YP and their parent/carer – if at all?

7. Did you feel ownership of the intervention, or did you perceive it as external to usual practice?

8. Have you had to make contact with other professionals to support the YP you've seen? With whom? For what reason?
   a. Have you taken any solution-focused practice / perspectives into those meetings? If so, how? And how did it go?

   (Probe: navigating referrals)

Experience of training and supervision

9. How did you feel about the training you received in delivering the intervention? *(Probe: sufficient length?)*

10. How was the supervision you received as part of the study? And any other support you might have received outside of supervision?

11. How did you find using the manual and handouts?

Impact on practice and professional development

12. Are there ways in which your practice has changed in working with young people presenting with self-harm / suicidal ideation since completing the training and delivering the intervention? If so, how?

13. Has your role as a SASH practitioner impacted your professional development, if so how?

Implementation of the intervention

14. If resources were available, would you choose to deliver an intervention like SASH beyond the end of the study?
    a. Why/ why not?

15. How could each of the TA, safety planning and solution-focused sessions fit within the context of delivering crisis care (across presentations, not just self-harm), thinking about interventions and pathways?

16. Which services / young people do you think this approach could be useful for beyond your team?
